# Ultraschalldiagnostik in der prähospitalen Notfallmedizin – brauchen wir eine standardisierte Ausbildung?

**DOI:** 10.1007/s00063-023-01045-4

**Published:** 2023-08-14

**Authors:** Christine Eimer, Ulf Lorenzen, Florian Reifferscheid, Nils Passau, Katharina Helzel, Alexander Schmuck, Stephan Seewald, Andrea Köser, Norbert Weiler, Holger Gässler, Björn Hossfeld, Matthias Gruenewald, Maximilian Feth

**Affiliations:** 1 Klinik für Anästhesiologie und Intensivmedizin, Universitätsklinikum Schleswig-Holstein, Campus Kiel, Christian-Albrechts-Universität Kiel, Kiel, Deutschland; 2 analytix GmbH, Institut für Qualitative Marktforschung und Datenanalyse, Kiel, Deutschland; 3 Klinik für Anästhesiologie und Notfallmedizin, Ameos-Klinik Eutin, Eutin, Deutschland; 4 Klinik für Anästhesiologie, Intensivmedizin, Notfallmedizin und Schmerztherapie, Bundeswehrkrankenhaus Ulm, Ulm, Deutschland; 5 Arbeitsgemeinschaft in Norddeutschland tätiger Notärzte, Lübeck, Deutschland; 6 Institut für Rettungs- und Notfallmedizin, Universitätsklinikum Schleswig-Holstein, Kiel, Deutschland

**Keywords:** Notfallsonographie, Notärztin/Notarzt, Point-of-Care-Ultraschall, Ultraschallausbildung, Prähospitale Bildgebung, Emergency sonography, Emergency medical services physician, Point-of-care ultrasound, Ultrasound training, Prehospital imaging

## Abstract

**Hintergrund:**

Zur Verbesserung der prähospitalen Notfalldiagnostik werden zahlreiche notarztbesetzte Rettungsmittel in Deutschland mit Ultraschallgeräten ausgestattet. Der Ausbildungsstand deutscher Notärzte in Notfallultraschallverfahren, die Verfügbarkeit und die Erfahrungen mit der prähospitalen Bildgebung sind bisher kaum evaluiert.

**Methoden:**

Bundesweite Onlinebefragung unter Notärzten hinsichtlich Ausbildung in und Erfahrungen mit prähospitaler Ultraschalldiagnostik.

**Ergebnisse:**

Im Studienzeitraum von 02/2022 bis 05/2022 wurden 1079 Teilnahmen an der Umfrage registriert. Es konnten 853 vollständige Fragebögen in die Auswertung eingeschlossen werden. 71,9 % der teilnehmenden Notärzte bewerten Point-of-Care-Ultraschall (POCUS) als sinnvolle Ergänzung der prähospitalen Notfalldiagnostik. 43,8 % der Teilnehmenden verfügt über eine standardisierte POCUS-Ausbildung. Teilnehmende mit zertifizierter POCUS-Ausbildung bewerten die eigene POCUS-Expertise besser als solche ohne eine entsprechende Ausbildung (*p* < 0,001). Die regelmäßige Anwendung von Ultraschall führt zu einer verbesserten Selbsteinschätzung der POCUS-Fähigkeiten.

**Diskussion:**

Die Mehrheit der Umfrageteilnehmer bewertet POCUS als Verbesserung der prähospitalen Notfalldiagnostik. Die Teilnahme an einer zertifizierten POCUS-Ausbildung sowie der regelmäßige Einsatz von Ultraschall führen zu einer verbesserten Bewertung der eigenen POCUS-Fähigkeiten.

**Zusatzmaterial online:**

Zusätzliche Informationen sind in der Onlineversion dieses Artikels (10.1007/s00063-023-01045-4) enthalten.

## Abstract (en)

**Background:**

Limited diagnostic capabilities represent an ongoing obstacle in out-of-hospital emergency settings. Prehospital deployment of ultrasound might reduce this particular diagnostic gap. So far, little is known about the availability and usage of ultrasound in emergency medical services (EMS) or about the level of education of EMS physicians regarding prehospital ultrasound (point-of-care ultrasound, POCUS).

**Methods:**

A nationwide survey was conducted among emergency physicians in Germany focusing on POCUS education and experience.

**Results:**

Between 02/2022 and 05/2022, 1079 responses were registered, of which 853 complete responses were analyzed. Of the emergency physicians, 71.9% consider POCUS beneficial for out-of-hospital diagnostics and 43.8% had participated in a certified POCUS training prior to the survey. The self-evaluation of POCUS skills among emergency physicians depended significantly on their participation in a certified training (*p* < 0.001) and frequent ultrasound routine (*p* < 0.001).

**Conclusion:**

The majority of participating emergency physicians in Germany consider POCUS to improve out-of-hospital diagnostic capabilities. Participation in a certified POCUS training and frequent use of ultrasound facilitated higher self-confidence in POCUS skills.

## Einleitung

Der Einsatz von Ultraschall (Point-of-Care-Ultraschall [POCUS]) eröffnet neue diagnostische Möglichkeiten in der prähospitalen Notfallmedizin [3]. Eine zertifizierte notfallsonographische Ausbildung ist bislang nicht in den entsprechenden Weiterbildungsordnungen verankert. Ziel dieser Studie ist die Evaluation des Ausbildungsstands der Notärzte in der Bundesrepublik Deutschland hinsichtlich POCUS sowie deren Selbsteinschätzung der eigenen POCUS-Kompetenz.

## Hintergrund und Fragestellung

Die prähospitale Notfallmedizin hat sich in den vergangenen Jahrzehnten im Zuge technischer Innovationen weiterentwickelt und stellt erweiterte Anforderungen an Notärzte. Die aktuelle Studienlage legt nahe, dass POCUS im Rahmen von Notfallsituationen zu einer früheren Diagnose kritischer Zustände, sichereren Indikationsstellungen bei invasiven Maßnahmen und verbesserten Krankenhauszuweisungen beiträgt [6, 12]. Ferner konnte gezeigt werden, dass der Einsatz von POCUS bei chirurgischen Patienten die prähospitalen Versorgungszeiten und die Dauer bis zur chirurgischen Intervention verkürzen kann [11, 18]. Ob sich ein ähnlicher Effekt auch bei nichttraumatologischen Patienten nachweisen lässt, bleibt Gegenstand der aktuellen Forschung. POCUS ist inzwischen Bestandteil gängiger Leitlinien und wird etwa für erfahrene Anwender in den aktuellen Empfehlungen des European Resuscitation Council zur Versorgung des Herz-Kreislauf-Stillstands empfohlen [20]. Gleichzeitig birgt der nichtfachgerechte Einsatz sonographischer Diagnostik am Einsatzort aber auch ein Risiko für Fehldiagnose, Therapie- und Zeitverzögerungen. Die Weiterbildungsordnungen zum Erwerb der Zusatzbezeichnung „Notfallmedizin“ unterscheiden sich in den Landesärztekammern. Voraussetzung zur Zulassung ist jedoch meist eine 24-monatige Weiterbildung in einem Gebiet der unmittelbaren Patientenversorgung sowie der Nachweis praktischer Fähigkeiten (z. B. Atemwegssicherung; [19]). Ein zertifiziertes Ausbildungsmodul zur prähospitalen Notfallsonographie existiert bis dato nicht. Ziel dieser Studie ist es, den POCUS-Ausbildungsstand und die -erfahrung von Notärztinnen und Notärzten zu evaluieren.

### Studiendesign

Bei der vorliegenden Studie handelt es sich um eine Onlinebefragung von Notärztinnen und Notärzten in der Bundesrepublik Deutschland. Die Studie wurde durch die Ethikkommission der Medizinischen Fakultät der Christian-Albrechts-Universität, Kiel begutachtet (ID: D543/21) sowie im Deutschen Register Klinischer Studien erfasst (DRKS, ID: DRKS00026763). Die Bewerbung der Umfrage erfolgte schwerpunktmäßig durch postalische Einladung aller Ärztlichen Leiter*Innen Rettungsdienst (ÄLRD) mit der Bitte um Bekanntmachung der Studie im Verantwortungsbereich. Zur Vereinfachung der Teilnahme waren dem Einladungsschreiben Sticker mit einem QR-Code zur direkten Weiterleitung zum Umfragemodul beigelegt. Wir baten die ÄLRD, diese Sticker den Notärzt*Innen in den notärztlichen Diensträumen oder Rettungsmitteln zugänglich zu machen. In Ergänzung hierzu erfolgte eine Bewerbung des Projekts auf notfallmedizinischen FOAM-Portalen (z. B. newspapers.eu, pin-up-docs.de), über die Internet- und Social-Media-Auftritte der Arbeitsgemeinschaft in Norddeutschland tätiger Notärzte (AGNN e.V.) sowie der Arbeitsgemeinschaft in Bayern tätiger Notärzte (abgn e. V.) sowie auf notfallmedizinisch relevanten Veranstaltungen (z. B. Jahrestagung der AGNN 2022). Die Onlineumfrage wurde mit der Software „LimeSurvey“ (LimeSurvey GmbH, Hamburg) umgesetzt. Der Fragebogen beinhaltet 18 Fragen, die entweder als „single-choice“-, „multiple-choice“- oder Likert-Skala-basierte Frage (Skalenniveau 0–10) konzipiert wurden. Der Fragebogen kann im Online-Supplement dieses Artikels eingesehen werden. Die Teilnahme an der Umfrage war vom 08.02.2022 bis zum 24.05.2022 möglich. Im Rahmen eines Disclaimers wurde zu Beginn der Umfrage sowohl eine Zustimmung zur Datenvereinbarung als auch zur Publikation der Studienergebnisse eingeholt. Die Teilnehmenden wurden darüber informiert, dass mittels Kontaktaufnahme mit dem Studienleiter jederzeit ein Widerruf der Umfrageteilnahme möglich war. Um einen Bias bei der Beantwortung des Fragebogens auszuschließen, waren die Ergebnisse weder vor der eigenen Teilnahme noch unmittelbar danach online abrufbar.

### Statistische Analyse

Statistische Analysen wurden mittels SPSS Statistics©, Version 27.0.1.0 (IBM Corp., 2020, Armonk, NY, USA) durchgeführt. Merkmale werden als Median sowie Interquartilsabstand dargestellt. Testungen auf Normalverteilung erfolgten mittels Shapiro-Wilk-Test. Testungen auf Gruppenunterschiede sowie Assoziationen von Einflussfaktoren erfolgten mittels Spearman-Rho-Test, Wilcoxon-Rank-Summentest oder Mann-Whitney-U-Test. Unterschiede mit einem α‑Fehler < 0,05 wurden als statistisch signifikant gewertet.

## Ergebnisse

**Abb. 1:**
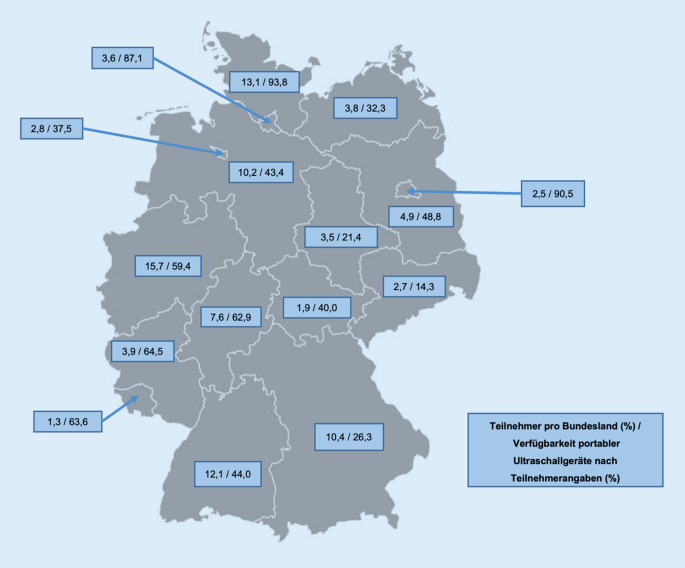
Verteilung der Teilnehmer sowie der Verfügbarkeit von portablen Ultraschallgeräten im Rettungsdienst in Prozent nach Bundesland geordnet entsprechend der Umfragerückmeldungen

Während des Studienzeitraums wurden 1079 Teilnahmen an der Umfrage registriert. 853 vollständige Datensätze konnten in die Analyse eingeschlossen werden, 226 (20,94 %) wurden aufgrund unvollständiger Daten ausgeschlossen. Tab. 1 fasst die demografische Beschreibung der Teilnehmer zusammen. Abb. 1 stellt die Verteilung der Teilnahmen nach Bundesland dar.

**Tab. 1:** Demografische Beschreibung des Studienkollektivs

Parameter	Anzahl (%)
**Fachgebiet**	
Anästhesiologie	587 (68,8)
Innere Medizin	99 (11,6)
Chirurgische Fachdisziplinen	68 (8,0)
Kinderheilkunde	5 (0,6)
Allgemeinmedizin	45 (5,3)
Andere Fachgebiete	49 (5,7)
*Versorgungslevel des Hauptarbeitgebers*	
Universitätsklinik	146 (17,1)
Maximalversorger	166 (19,5)
Schwerpunktversorger	166 (19,5)
Grund- und Regelversorger	212 (24,9)
Rehabilitationsklinik	4 (0,5)
Niederlassung	69 (8,1)
Ausschließliche Tätigkeit im Notarztdienst	82 (9,6)
Andere Tätigkeiten (z. B. Amtsarzt)	8 (0,9)
**Erfahrung als Notarzt**	
< 1 Jahre	68 (8,0)
1–2 Jahre	96 (11,3)
3–5 Jahre	153 (17,9)
6–10 Jahre	170 (19,9)
> 10 Jahre	366 (42,9)
**Geschätzte Anzahl absolvierter Notarzteinsätze binnen 2 Jahren vor Umfrageteilnahme**	
< 100	139 (16,3)
100–250	279 (32,7)
251–500	265 (31,1)
501–1000	127 (14,9)
> 1000	43 (5,0)
**Rettungsmittel**	
Bodengebundener Primäreinsatz	714 (83,7)
Luftgebundener Primäreinsatz	113 (13,2)
Sekundäreinsätze (z. B. ITW) und andere	26 (3,0)
**Verfügbarkeit portabler Ultraschallgeräte im Notarztdienst**	444 (52,1)
**Ultraschalluntersuchungen/Monat im klinischen Alltag**	
< 10	298 (34,9)
11–50	374 (43,8)
51–100	111 (13,0)
> 100	70 (8,2)
**Teilnahme an einer zertifizierten Ultraschallausbildung**	*374 (43,8)*

52,1 % aller teilnehmenden Notfallmedizinerinnen und Notfallmediziner gaben an, im Notarztdienst ein Ultraschallgerät zur Verfügung zu haben (bundeslandabhängig 14,3–93,8 %). Die Teilnehmer stammten hauptsächlich aus der Anästhesiologie (68,8 %) oder der inneren Medizin (11,6 %). Die Mehrheit der teilnehmendenÄrztinnen und Ärzte verfügt über mehr als 10 Jahre Erfahrung im Notarztdienst (42,9 %) und gibt eine Einsatzroutine von 100–500 Notarzteinsätzen in einem Zeitintervall von 2 Jahren vor Teilnahme an der Umfrage an (63,8 %). Der Einsatz erfolgt meist im bodengebundenen Rettungsdienst (83,7 %).

### Innerklinischer und prähospitaler Einsatz von Ultraschall

71,9 % der Teilnehmer erachten den Einsatz von POCUS in der prähospitalen Notfallmedizin als zielführend und hilfreich. Im jeweiligen klinischen Alltag geben die Teilnehmer an, pro Monat 0–10 (34,9 %), 11–50 (43,8 %), 51–100 (13,0 %), > 100 (8,2 %) Ultraschallanwendungen inklusive POCUS durchzuführen. Die Teilnehmer bewerten ihre eigenen POCUS-Fähigkeiten im Rahmen gängiger Ultraschallalgorithmen am besten im Rahmen des Protokolls Extended Focused Assessment with Sonography for Trauma (72,1 %). 43,8 % der teilnehmenden Notfallmediziner verfügen über eine standardisierte POCUS-Ausbildung entsprechend den Regularien der Deutschen Gesellschaft für Ultraschall in der Medizin (DEGUM).

### Selbsteinschätzung der POCUS-Fähigkeiten

Die Erfahrung in Notfallultraschalluntersuchungen wurde durch das Studienkollektiv auf der Likert-Skala von 0–10 mit einem Median von 7 (IQR 4–8) angegeben. 4,3 % der Teilnehmer geben keine Erfahrung in Notfallultraschallverfahren (Likert-Skala = 0) und 8,2 % bestmögliche POCUS-Erfahrungen (Likert-Skala = 10) an. Organbezogen bewerten die Teilnehmer die eigenen POCUS-Fähigkeiten am besten für Untersuchungen der Lunge/Pleura (Median 7, IQR 5–9) gefolgt von Abdomen (Median 6, IQR 4–8) und Herz (Median 5, IQR 3–7).

### Einflussfaktoren auf die Selbsteinschätzung der POCUS-Fähigkeiten

**Abb. 2:**
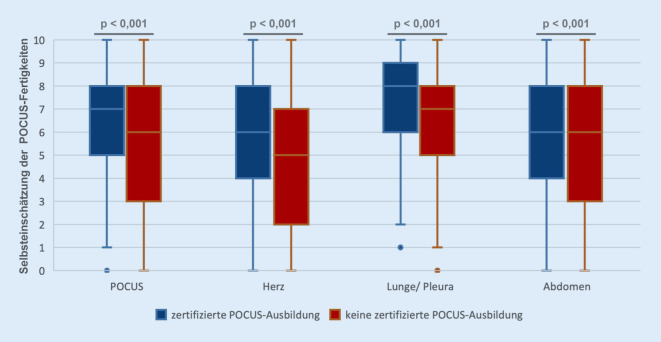
Einfluss eines zertifizierten POCUS-Trainings auf die Selbsteinschätzung von POCUS-Fähigkeiten von Notärzten. *Boxplot*: Median, horizontaler Marker innerhalb der Box; „range“ der Box von der 25. bis zur 75. Perzentile. „Error bars“ stellen die Minimal- und Maximalausprägungen dar. *POCUS* Point-of-Care-Ultraschall

Die Teilnahme an einer zertifizierten Ultraschallausbildung entsprechend der DEGUM-Regularien führte zu einer signifikant besseren Bewertung der eigenen POCUS-Kompetenzen im Allgemeinen (*p* < 0,001) sowie organbezogen für Herz, Lunge (beide *p* < 0,001) und Abdomen (*p* = 0,002, s. Abb. 2). Darüber hinaus konnte ein statistisch signifikanter Zusammenhang zwischen der Anzahl monatlicher Ultraschalluntersuchungen im klinischen Alltag und der Selbsteinschätzung der allgemeinen und organspezifischen POCUS-Fähigkeiten gezeigt werden (jeweils *p* < 0,001). Die Einsatzhäufigkeit im Notarztdienst korreliert statistisch signifikant mit der Einschätzung der allgemeinen POCUS-Fähigkeiten (*p* = 0,021). Außerdem konnte ein statistisch signifikanter Zusammenhang zwischen der Erfahrung als Notarzt gemessen in Jahren der Teilnahme am Notarztdienst und der Selbsteinschätzung der allgemeinen POCUS-Fähigkeiten (*p* = 0,045) sowie im Lungen- und Pleuraultraschall (*p* = 0,001) gezeigt werden. Tab. 2 fasst wesentliche Einflussfaktoren auf die Selbsteinschätzung von POCUS-Fähigkeiten der Umfrageteilnehmer zusammen.

**Tab. 2:** Einflussfaktoren auf die Selbsteinschätzung von Notärzten in Point-of-Care-Ultraschall (*POCUS*). (Median; Interquartilsabstand)

						*p*
**Teilnahme an einer zertifizierten Ausbildung**	**Ja**	*–*	**Nein**	**–**		**–**
POCUS	7; 5–8		6; 3–8			*<* *0,001*
Herz	6; 4–8		5; 2–7			*<* *0,001*
Lunge/Pleura	8; 6–9		7; 5–8			*<* *0,001*
Abdomen	6; 4–8		6; 3–8			*0,002*
**Geschätzte Anzahl von Notarzteinsätzen innerhalb von 2 Jahren vor Umfrageteilnahme**	**<** **100**	**100–250**	**251–500**	**501–1000**	**>** **1000**	**–**
POCUS	6; 3–8	6; 4–8	7; 4–8	7; 4–8	7; 6–8	*0,021*
Herz	5; 3–7	5; 3–7	5; 3–7	5; 3–8	6; 4–8	0,579
Lunge/Pleura	7; 5–8	7; 5–8	7; 6–9	8; 5–9	8; 6–9	0,383
Abdomen	6; 4–7	6; 4–8	6; 3–8	7; 4–8	6; 5–9	0,181
**Klinische Ultraschallroutine**	**<** **10**	**10–50**	**51–100**	**>** **100**	**–**	**–**
POCUS	4; 2–6	7; 5–8	8; 7–9	9; 8–10	–	*<* *0,001*
Herz	3; 1–6	6; 4–7	7; 5–8	7; 6–9	–	*<* *0,001*
Lunge/Pleura	6; 3–8	8; 6–9	8; 7–9	9; 8–9	–	*<* *0,001*
Abdomen	4; 2–6	6; 4–8	7; 6–9	9; 7–10	–	*<* *0,001*
**Notärztliche Erfahrung (Jahre)**	*<* **1**	**1–2**	**3–5**	**6–10**	**>** **10**	**–**
POCUS	6; 4–7	6; 4–7	6; 5–8	7; 5–8	7; 3–8	*0,045*
Herz	5; 3–7	5; 3–7	5; 3–8	6; 4–7	5; 2–7	0,118
Lunge/Pleura	7; 5–8	7; 5–8	8; 7–9	8; 6–9	7; 5–9	*0,001*
Abdomen	6; 4–7	6; 4–8	6; 3–7	6; 4–8	6; 3–9	0,303

## Diskussion

Die Mehrzahl der teilnehmenden Notärzte erachtet den Einsatz von POCUS in der prähospitalen Notfallmedizin als sinnvoll und gewinnbringend. Die Einschätzung der eigenen POCUS-Fähigkeiten wird durch die Teilnahme an einer zertifizierten Ausbildung sowie durch eine regelmäßige Anwendung von Ultraschallverfahren in der klinischen Praxis signifikant verbessert. Darüber hinaus führte ein häufiger Einsatz im Notarztdienst zu einer besseren Selbsteinschätzung der POCUS-Expertise im Allgemeinen ohne sich jedoch in der Einschätzung der organspezifischen POCUS-Untersuchungen widerzuspiegeln. Diese Studie stellt die bisher umfassendste Evaluation von POCUS-Erfahrungen, Ausbildungen und Selbsteinschätzung der Notfallultraschallverfahren unter Notärzten im deutschsprachigen Raum dar [9].

Die Verfügbarkeit portabler Ultraschallgeräte im Rettungsdienst erscheint heterogen. Je nach Bundesland geben 14,3–93,8 % der Teilnehmer an, im Notarztdienst ein Ultraschallgerät zur Verfügung zu haben. Im Jahr 2019 hat die DEGUM als Ultraschallfachgesellschaft die Vorhaltung von Ultraschallgeräten auf notarztbesetzten Rettungsmitteln empfohlen [5]. Sicherlich ist die Häufigkeit von POCUS-Geräten auf diesen Rettungsmitteln in der vorliegenden Arbeit eine Momentaufnahme, sie zeigt jedoch, das POCUS im Rettungsdienst aktuell nicht flächendeckend verfügbar ist. Eine Empfehlung der für die prähospitale Notfallmedizin zuständigen Fachgesellschaften wäre wünschenswert.

Aktuell werden verschiedene zertifizierte POCUS-Ausbildungen, wie etwa der Kurs „Basisausbildung Notfallsonographie“ der DEGUM, auf dem deutschsprachigen Markt angeboten [13]. Die Weiterbildungen umfassen neben theoretischen Grundlagen in aller Regel Hands-on-Anteile mit engem Ausbilder-Teilnehmer-Verhältnis und werden mit einer Prüfung abgeschlossen [8]. Die teilnehmenden Ärzte*innen haben an zertifizierten Kursen zur Notfallsonographie der DEGUM teilgenommen. Ein spezielles zertifiziertes Ausbildungsmodul zur prähospitalen Notfallsonographie, das rettungsdienstspezifische Besonderheiten adressiert, existiert bis dato nicht, wäre jedoch wünschenswert. Notärzte, die an einer Ausbildung gemäß DEGUM teilgenommen haben, schätzen entsprechend der vorliegenden Ergebnisse die eigene POCUS-Expertise besser ein als solche ohne ein zertifiziertes Training. Dies deckt sich mit Ergebnissen von Krogh et al, die eine Verbesserung der Untersuchungsqualität von Notärzten nach einer strukturierten Einweisung in POCUS zeigen konnten [10]. Neben der einmaligen Überprüfung der POCUS-Leistung am Kursende gibt es für Notärzte keine wiederkehrende Beurteilung der eigenen Ultraschallkompetenz. Nichtsdestotrotz legen die Ergebnisse dieser Studie nahe, dass die Integration eines Notfallsonographiecurriculums in die Ausbildung der Notärzte für die erfolgreiche Implementierung von POCUS in der prähospitalen Notfallmedizin sinnvoll wäre.

Neben der Ausbildung bestätigen die Ergebnisse dieser Studie die regelmäßige Anwendung von Ultraschall im ärztlichen Alltag als positiven Einflussfaktor auf die Selbsteinschätzung der POCUS-Expertise. Wie auch beim Atemwegsmanagement in Notfallsituationen scheint der häufige Einsatz der Technik die Anwendersicherheit in Notfällen zu verbessern [7, 17]. Entsprechend scheint eine regelmäßige Anwendung von Ultraschall im klinischen Alltag Grundlage für den erfolgreichen Einsatz von POCUS in der Notfallmedizin zu sein [4], da insbesondere auch in Notfallsituationen mit erschwerten Schallbedingungen zu rechnen ist.

Bislang sind keine flächendeckenden Systeme zur Qualitätssicherung von prähospitalem POCUS in Deutschland etabliert. Solche Maßnahmen werden jedoch z. B. durch das American College of Emergency Physicians als fortlaufende Beurteilungen von 5–10 % der durchgeführten Notfallultraschalluntersuchungen empfohlen [1]. Die Implementation von Qualitätssicherungsmaßnahmen könnte eine hilfreiche Ergänzung zu einer qualitativ hochwertigen Verbesserung der prähospitalen Notfalldiagnostik darstellen [14, 15]. Solche Maßnahmen und auch der mögliche Einsatz von künstlicher Intelligenz zur Verbesserung der prähospitalen Notfalldiagnostik sollten wissenschaftlich begleitet für eine sinnvolle Anwendung im Rettungsdienst evaluiert werden.

Die wesentlichen Limitationen dieser Arbeit bestehen im Selektionsbias der Teilnehmer sowie in der unklaren Repräsentativität des Teilnehmerkollektivs. Ein Selektionsbias könnte hierbei insofern bestehen, als das POCUS-affine Notärzte sich eher zur Teilnahme an der Umfrage aufgerufen sahen als solche, die POCUS zurückhaltend gegenüberstehen. Daraus ergibt sich möglicherweise auch die hohe Anzahl an Teilnehmern, die POCUS als sinnvolle Ergänzung der prähospitalen Notfallmedizin erachten. Der Effekt eines solchen Bias könnte durch ein Vergleichskollektiv gemindert werden, das unabhängig von der Fragestellung eine große Anzahl von in Deutschland tätigen Notärzten erfasst, demografisch beschreibt und somit Rückschlüsse auf den demografischen Querschnitt der aktuellen Arbeit ermöglicht. Eine solche Arbeit ist den Autoren dieser Studie bisher nicht bekannt. Folglich können Studienergebnisse wie etwa die positive Bewertung von POCUS nicht extern durch eine Vergleichskohorte validiert werden. Darüber hinaus stellt die Fokussierung der Umfrage auf die DEGUM-zertifizierte POCUS-Ausbildung eine Schwäche dieser Arbeit dar [2]. Neben dem DEGUM-zertifizierten Kurs bietet beispielsweise die Deutsche Gesellschaft für Anästhesiologie und Intensivmedizin ein gleichwertiges Training an [16]. Die Auswahl des interdisziplinären DEGUM-Kurses als Referenzausbildung erfolgte aufgrund der Vielzahl unterschiedlicher Fachgebiete des angesprochenen Teilnehmerkollektivs. Eine namentliche Integration weiterer zertifizierter POCUS-Ausbildungen hätte jedoch zu einer höheren Rate an zertifiziert ausgebildeten Teilnehmern führen können. Letztlich erfasst diese Arbeit die Selbsteinschätzung der POCUS-Expertise von Notärzten und nicht die tatsächliche Qualität der Ultraschalluntersuchung im Notfalleinsatz. Obwohl wünschenswert erscheint eine solche Erfassung der POCUS-Einsätze in der prähospitalen Notfallmedizin im gesamten Bundesgebiet vor allem aufgrund der nicht flächendeckenden Verfügbarkeit von Ultraschallgeräten und der in vielen Bereichen fehlenden digitalen Datenerfassung schwierig umzusetzen.

## Fazit für die Praxis

- Die Mehrheit der Teilnehmer erachtet den Einsatz von POCUS in der prähospitalen Notfallmedizin als zielführend und hilfreich.
- Etwa die Hälfte aller Umfrageteilnehmer verfügt über eine standardisierte POCUS-Ausbildung entsprechend den Regularien der Deutschen Gesellschaft für Ultraschall in der Medizin (DEGUM).
- Eine strukturierte Ausbildung sowie eine regelmäßige Anwendung von Ultraschall im klinischen Alltag verbessern die POCUS-Kompetenz.
- Die Aufnahme eines zertifizierten Nachweises über notfallsonographische Diagnostik in die Weiterbildungsordnung zur Zusatzbezeichnung „Notfallmedizin“ sowie die Forderung nach einem regelmäßigen Einsatz von Ultraschallverfahren im klinischen Alltag eines Notarztes erscheint sinnvoll.

## Supplementary Information

Fragebogen

## Abkürzungen

Agbn: Arbeitsgemeinschaft in Bayern tätiger Notärzte und Notärztinnen
AGNN: Arbeitsgemeinschaft in Norddeutschland tätiger Notärzte
DEGUM: Deutsche Gesellschaft für Ultraschall in der Medizin
DGAI: Deutsche Gesellschaft für Anästhesiologie und Intensivmedizin
DRKS: Deutsches Register Klinischer Studien
E‑FAST: Extended Focused Assessment with Sonography in Trauma
FATE: Focus Assessed Transthoracic Echocardiography
IQR: Interquartilsabstand
POCUS: Point-of-Care-Ultraschall
pUSD: Portables Ultraschallgerät
RUSH: Rapid Ultrasound in Shock and Hypotension
