# Supplementary material for: Ultrasound diagnostics in prehospital emergency care—do we need a standardized educational approach?
Source: Med Klin Intensivmed Notfmed. 2023 Aug 14;119(4):309–15. [Article in German] doi: 10.1007/s00063-023-01045-4 (PMC11058608; doi:10.1007/s00063-023-01045-4)

1. **In welchem Bundesland sind Sie notärztlich vorwiegend tätig?**

Bitte wählen Sie die zutreffenden Antworten aus:

 Bayern

 Baden-Württemberg

 Berlin

 Brandenburg

 Bremen

 Hamburg

 Hessen

 Mecklenburg-Vorpommern

 Niedersachsen

 Nordrhein-Westfalen

 Rheinland-Pfalz

 Saarland

 Sachsen

 Sachsen-Anhalt

 Schleswig-Holstein

 Thüringen

1. **In welchem Fachgebiet arbeiten Sie?**

Bitte wählen Sie die zutreffenden Antworten aus:

 Allgemeinmedizin

 Anästhesiologie

 Chirurgie

 Innere Medizin

 Kinderheilkunde

 Sonstiges

1. **Wo arbeiten Sie?**

Bitte wählen Sie eine Antwort:

 ausschließlich als Notarzt/Notärztin

 Grund- und Regelversorger

 Maximalversorger

 Praxis/Niederlassung

 Reha-/Privatklinik

 Schwerpunktversorger

 Universitätsklinikum

 Sonstiges:

1. **Wie lange sind Sie bereits als Notarzt/Notärztin tätig?**

Bitte wählen Sie die zutreffende Antwort aus:

 < 1 Jahr

 1-2 Jahre

 3 - 5 Jahre

 6 - 10 Jahre

 > 10 Jahre

**5. Wie viele Einsätze haben Sie schätzungsweise in den letzten 2 Jahren absolviert?**

Bitte wählen Sie die zutreffende Antwort aus

 < 100
 100 – 250

 251 – 500

 501 – 1000

 > 1000

1. **Auf welchem Rettungsmittel werden Sie vorwiegend eingesetzt?**

Bitte wählen Sie die zutreffende Antwort aus:

 ITW

 NAW

 NEF

 RTH/ITH

 VEF

1. **Wird auf Ihrem Rettungsmittel ein Ultraschallgerät vorgehalten?**

Bitte wählen Sie die zutreffenden Antworten aus:

 Ja
 nein
 ich besitze ein eigenes portables Ultraschallgerät

1. **Für wie sinnvoll halten Sie den Einsatz von Ultraschall in der Notfallmedizin? (0= garnicht sinnvoll; 10= sehr sinnvoll)**

Bitte wählen Sie die zutreffende Antwort aus:

**0 1 2 3 4 5 6 7 8 9 10**

1. **Wie häufig führen Sie Ultraschalluntersuchungen pro Monat durch?**

Bitte wählen Sie die zutreffende Antwort aus:

 < 10
 10 – 50

 51 – 100

 > 100

1. **Können Sie bestimmte Untersuchungsgänge nach Ihrem Ermessen sicher anwenden (E-FAST und/oder FATE und/oder RUSH und/oder vergleichbare)?**

Bitte wählen Sie die zutreffende Antwort aus:

 ja

 nein

1. **Welchen Untersuchungsgang beherrschen Sie (Mehrfachauswahl möglich)?**

Beantworten Sie diese Frage nur, wenn folgende Bedingungen erfüllt sind:
Können Sie bestimmte Untersuchungsgänge nach Ihrem Ermessen sicher anwenden (E-FAST und/oder FATE und/oder RUSH und/oder vergleichbare)?

Bitte wählen Sie die zutreffenden Antworten aus:

 E-FAST

 FATE

 RUSH

 Sonstiges:

1. **Haben Sie an dem DEGUM-zertifizierten Kurs „Notfallsonographie“ teilgenommen?**

Bitte wählen Sie die zutreffenden Antworten aus:

 ja

 nein

1. **Wie schätzen Sie Ihre Erfahrung mit Ultraschalluntersuchungen in einer Notfallsituation ein? (0= nicht vorhanden; 10= vollkommen sicher)**

Bitte wählen Sie die zutreffende Antwort aus:

**0 1 2 3 4 5 6 7 8 9 10**

1. **Wie schätzen Sie Ihre Erfahrung mit ultraschallgesteuerter Regionalanästhesie ein? (0= nicht vorhanden; 10= vollkommen sicher)**

Bitte wählen Sie die zutreffende Antwort aus:

**0 1 2 3 4 5 6 7 8 9 10**

1. **Wie schätzen Sie Ihre Erfahrung mit Ultraschall des Herzens ein? (0= nicht vorhanden; 10= vollkommen sicher)**

Bitte wählen Sie die zutreffende Antwort aus:

**0 1 2 3 4 5 6 7 8 9 10**

1. **Wie schätzen Sie Ihre Erfahrung mit Ultraschall der Lunge/Pleura ein? (0= nicht vorhanden; 10= vollkommen sicher)**

Bitte wählen Sie die zutreffende Antwort aus:

**0 1 2 3 4 5 6 7 8 9 10**

1. **Wie schätzen Sie Ihre Erfahrung mit Ultraschall des Abdomens ein? (0= nicht vorhanden; 10= vollkommen sicher)**

Bitte wählen Sie die zutreffende Antwort aus:

**0 1 2 3 4 5 6 7 8 9 10**

1. **Wurde von Ihrem Rettungsdienst-Träger/ notfallmedizinischen Arbeitgeber eine Ultraschallschulung angeboten?**

Bitte wählen Sie die zutreffende Antwort aus:

 ja, ich habe teilgenommen

 ja, ich habe aber nicht teilgenommen

 nein, es wurde keine Schulung angeboten

 ich weiß es nicht


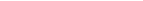

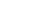

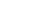

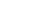

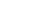

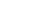

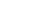

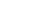

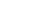

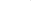

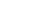

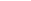

Supplement: Supplementary file 1 — Fragebogen [file 63_2023_1045_MOESM1_ESM.docx]
